# Burned-Out Testicular Tumors in Adolescents: Clinical Aspects and Outcome

**DOI:** 10.3389/fped.2021.688021

**Published:** 2021-08-25

**Authors:** Giorgio Persano, Alessandro Crocoli, Maria Debora De Pasquale, Raffaele Cozza, Rita Alaggio, Francesca Diomedi Camassei, Federico Beati, Pierluigi Di Paolo, Cristina Martucci, Alessandro Inserra

**Affiliations:** ^1^Surgical Oncology–General and Thoracic Surgery Unit, Department of Surgery, Bambino Gesù Children Hospital IRCCS, Rome, Italy; ^2^Oncohematology Unit, Department of Oncohematology and Gene Therapy, Bambino Gesù Children Hospital IRCCS, Rome, Italy; ^3^Pathology Unit, Department of Laboratories, Bambino Gesù Children Hospital IRCCS, Rome, Italy; ^4^Radiology Unit, Department of Diagnostic Imaging, Bambino Gesù Children Hospital IRCCS, Rome, Italy

**Keywords:** germ cell tumor, children, burned out germ cell tumor, adolescents, testis

## Abstract

**Purpose:** Testicular germ cell tumors are the fourth most common neoplasm in adolescents, accounting for 8% of all tumors in the age group 15–19 years. On rare instances, the primary testicular lesion is not clinically or radiologically evident while nodal or visceral metastases represent the clinical manifestations of the disease. This phenomenon is described as “burned-out testicular tumor.” In this paper, the authors report a single-institution experience with burned-out testicular tumors in adolescents and discuss their clinical implications.

**Patients and Methods:** All the patients diagnosed with metastatic testicular germ cell tumors at Bambino Gesù Children Hospital between January 1, 2010, and June 30, 2020, were included in the study. Patients were categorized into two groups: “primary testicular” and “burned out.” All the patients were staged and treated according to the AIEOP–TCGM 2004 protocol.

**Results:** Eleven patients were classified as “primary testicular,” and five patients were classified as “burned out.” “Burned-out” tumors were associated with the presence of systemic symptoms compared to “primary testicular” tumors (80 vs. 0%; *p* = 0.0027) and higher aFP, hCG, and LDH levels (*p* < 0.00001). The “burned-out” population had a statistically significant higher incidence of relevant toxicity than the “primary testicular” population (80 vs. 18%; *p* = 0.0357) and a worse outcome in terms of both mean overall survival (15 vs. 43 months; *p* = 0.0299) and mean event-free survival (12 vs. 38 months; *p* = 0.0164).

**Conclusion:** “Burned-out” testicular tumors seem to be a well-distinct clinical entity with a high treatment-related toxicity and poor prognosis. Further studies are needed to clarify the “burned-out phenomenon” and to identify more effective therapeutic strategies for these patients.

## Introduction

Testicular germ cell tumors are the fourth most common neoplasm in adolescents, accounting for 8% of all tumors in the age group 15–19 years (1) with an estimated prevalence in Europe of 24.5 cases per million inhabitants (2).

The prognosis of germ cell tumors is generally excellent, even though a subset of patients, i.e., patients older than 11 years, with elevated alfa-fetoprotein levels at diagnosis, extragonadal primary tumors, non-germinomatous tumors, and stage III and IV disease, have a worse outcome and therefore need an intensified treatment (3–6).

On rare instances, the primary testicular lesion is not clinically or radiologically evident while nodal or visceral metastases remain viable and represent the clinical manifestations of the disease (7). In these patients, the only histological evidence of a testicular origin of the tumor is a characteristic pattern of testicular scarring with hematoxylin staining bodies that contain calcium and DNA, often associated with peripheral atrophy and intratubular malignant germ cells (8). This phenomenon is described as “burned-out testicular tumor” or “spontaneously regressed testicular tumor” (8–11). Burned-out testicular tumors have been extensively described in the literature from a histological point of view; on the other hand, the clinical aspects of testicular burned-out tumors have not been fully characterized to date.

In the present paper, the authors report a single-institution experience with burned-out testicular tumors in adolescents and discuss their clinical implications.

## Materials and Methods

All the patients diagnosed with metastatic testicular germ cell tumors at Bambino Gesù Children Hospital between January 1, 2010, and June 30, 2020, were included in the study.

**Figure 1:**
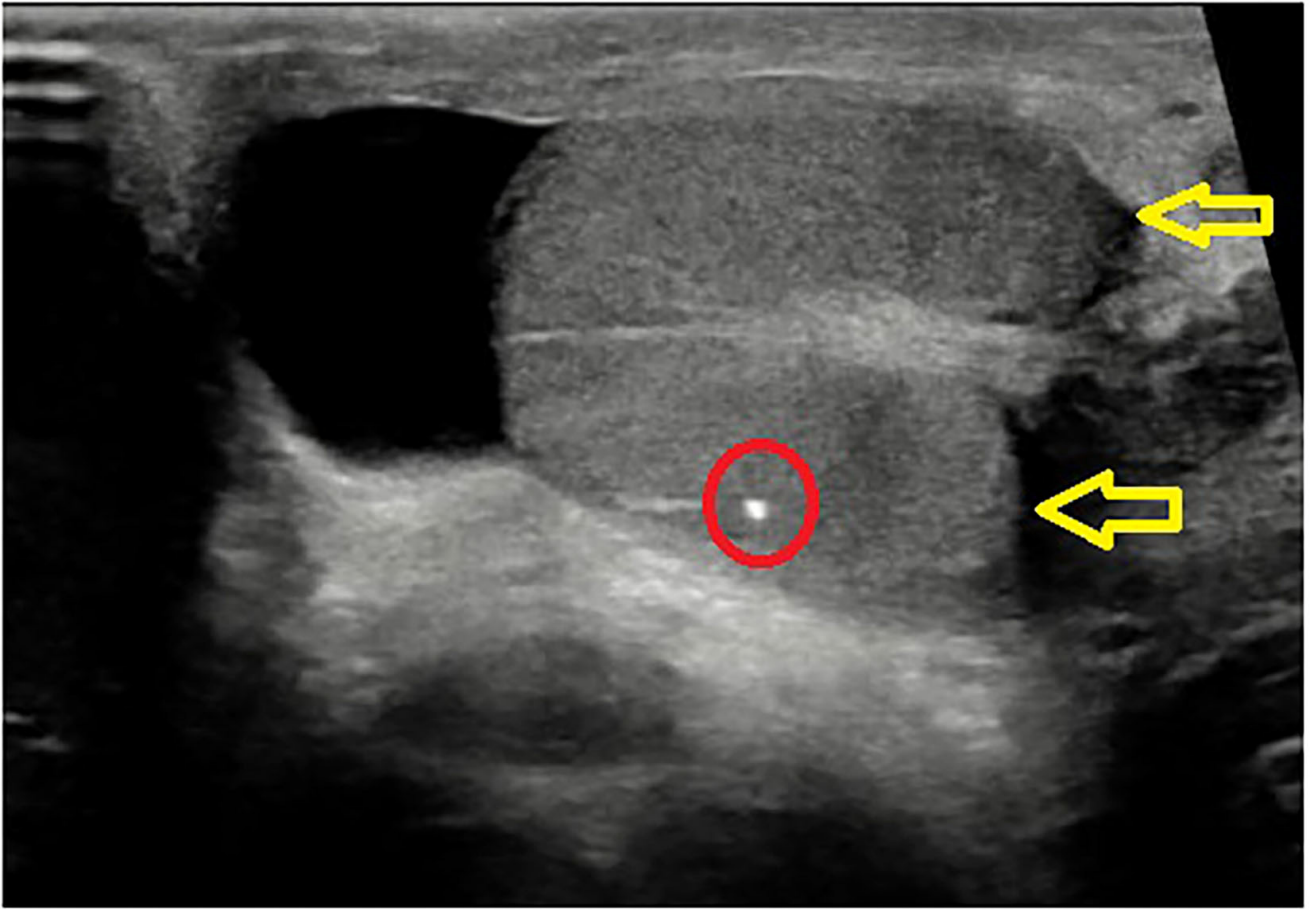
Burned out echographic appearance: scrotal ultrasound showing testicle with microcalcifications and hypoechogenic nodule.

The clinical notes of all the patients with stage III and IV tumors were retrospectively reviewed and categorized in two broad groups: “primary testicular” patients, i.e., patients who presented with testicular pain or testicular enlargement at initial diagnosis and sonographic evidence of testicular mass, and “burned-out” patients, i.e., patients who had no testicular symptoms at initial presentation and non-specific findings at ultrasound, such as microcalcification (Figure 1) and parenchymal heterogeneity in one or both testes. At the time of admission, all the patients were staged and treated according to the AIEOP–TCGM 2004 protocol (5). Hepatic, renal, pulmonary, and cardiac function were evaluated before the initiation of treatment. All the patients received first-line treatment with PEB chemotherapy regimen (Cisplatin 25 mg/m^2^/day iv for 4 days, Etoposide 100 mg/m^2^/day iv for 4 days, Bleomycin 15 UI/m^2^ on day 2), three courses for stage III disease, and four courses for stage IV disease; indications for resection of secondary lesions after chemotherapy were considered for individual cases.

Seven variables have been examined: age at diagnosis, clinical presentation, serum markers level, stage, primary histology, toxicity, and outcome. Follow-up time was calculated from the time of diagnosis until the time of last follow-up visit with a cutoff in October 2020. Statistical analyses were performed using Prism 9.0.0.121 (GraphPad Software, Inc., San Diego, CA). A value of *p* < 0.05 was considered statistically significant for each analysis.

### Age at Diagnosis

Mean age at diagnosis has been calculated separately in the two groups and data have been compared using Student's *t*-test.

### Clinical Presentation

The presence at initial diagnosis of “mass effect” symptoms, defined as extra-testicular palpable mass or symptoms related to compression due to metastatic mass, or “systemic” symptoms, defined as fever > 38°C, weight loss, and deep venous thrombosis, was searched for in the clinical notes of each patient in both groups. Data were compared using Fisher's test.

### Serum Markers Level

Alpha-fetoprotein (aFP), human chorionic gonadotropin (hCG), and lactate dehydrogenase (LDH) levels were measured at initial presentation in all the patients. Mean and standard deviation for each serum marker were calculated separately in the two groups and data were compared using Student's *t*-test.

### Stage

In each group, patients were divided into two subgroups according to the stage of the disease, i.e., stage III and IV. For stage IV subgroup, patients with extra-pulmonary visceral metastases were analyzed separately on the basis of the evidence of a distinct worse prognosis in adult population (12). Data were compared using Fisher's test.

### Primary Histology

All the patients had their diagnosis confirmed by histological examination of the affected testis or a specimen from the metastatic mass. Patients were classified according to the AIEOP–TCGM 2004 protocol as mature teratoma, immature teratoma, yolk sac tumor, choriocarcinoma, embryonal carcinoma, seminoma, or mixed histology (5). Data were compared using Fisher's test.

### Toxicity

Patients were defined as having experienced “relevant toxicity” during treatment if they had at least one episode of grade 3 or higher toxicity in at least one apparatus as defined in the “Common Terminology Criteria for Adverse Events (CTCAE) version 5.0” (13). Data were compared using Fisher's test.

### Outcome

Patient's outcome has been registered at the time of last follow-up visit as “remission” if the patient had no clinical, radiological, or serological evidence of disease; “progression” if the patient had clinical, radiological, or serological signs of disease; or “death” if the patient had passed away. Event-free survival (EFS) was calculated from the date of initial treatment to the date of whichever came first among progression, death, and last follow-up visit. Overall survival (OS) was measured from the date of initial diagnosis to the date of death or last follow-up visit. Survival was analyzed with Kaplan–Meier plots and the log-rank test. A value of *p* < 0.05 was considered statistically significant.

## Results

In the examined period, 16 patients were diagnosed with stage III and IV testicular germ cell tumors at our institution and all were included in the present study.

Eleven patients were classified as “primary testicular:” four patients (36%) had right testicular tumor and seven (64%) had tumor in the left testis. Five patients were classified as “burned out:” two patients (40%) had right-side tumor, two had left-side tumor (40%), and in one patient (20%), the location of the primitive tumor could not be determined.

None of the patients had pre-existing comorbidities.

### Age at Diagnosis

“Burned-out” patients were significantly older (mean age 17 years 7 months, range 15 years 10 months to 19 years 11 months) than “primary testicular” patients (mean age 15 years, range 0 years 6 months to 24 years 0 months, *p* < 0.00001).

### Clinical Presentation

None of the patients classified as “primary testicular” presented with “mass related” symptoms or “systemic” symptoms.

All the “burned-out” patients presented with symptoms related to mass effect due to metastases. Four “burned-out” patients (80%) presented with systemic symptoms (i.e., weight loss in three patients, fever > 38°C and deep venous thrombosis in two patients). The association between “burned-out” tumors and systemic symptoms was statistically significant (*p* = 0.0027).

### Serum Markers Level

aFP, hCG, and LDH levels were significantly higher in the “burned-out” population than in “primary testicular” patients: mean aFP 2,215 ng/ml (range 2–10,997 ng/ml) vs. 979 ng/ml (range 2.05–4,604.3 ng/ml), *p* < 0.00001; mean hCG 14,831 mIU/ml (range 2–73,564.5 mIU/ml) vs. 530 mIU/ml (range 2–1,863.5 mIU/ml), *p* < 0.00001; mean LDH 1,558 IU/L (range 372–4,052 IU/L) vs. 424 IU/L (range 238–620 IU/L), *p* < 0.00001.

### Stage

In the “primary testicular” population, six patients (55%) were stage III and five patients (45%) were stage IV; one patient (9%) had extrapulmonary visceral metastases in the central nervous system.

**Figure 2:**
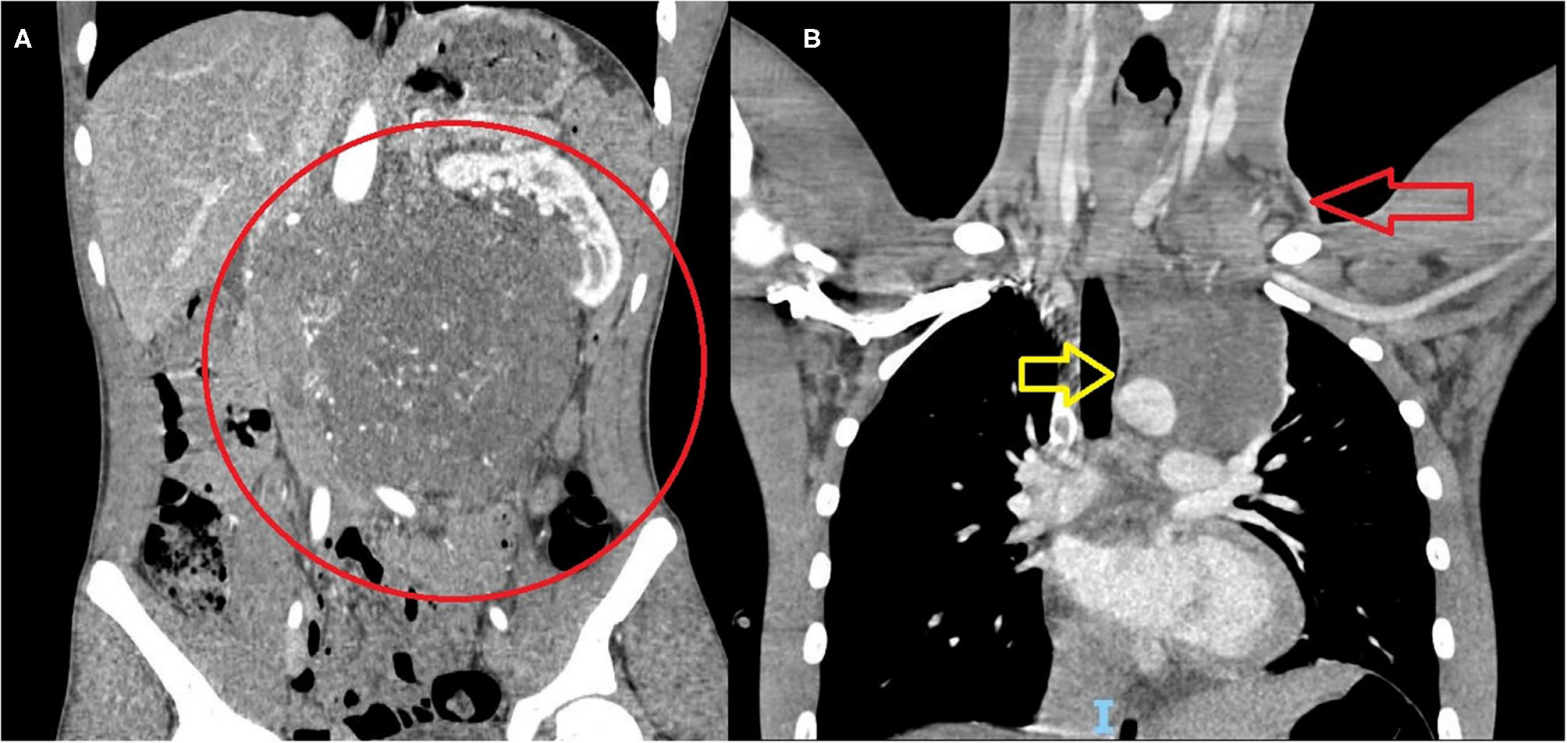
Massive metastatic involvement of retroperitoneal **(A)** and mediastinal **(B)** lymph-nodes.

In the “burned-out” population, one patient (20%) was stage III and four patients (80%) were stage IV; three patients (60%) had extrapulmonary visceral metastases, including liver (three patients), bones (two patients), and central nervous system (one patient) (Figure 2).

There was no statistically significant difference in the stage distribution between the two groups (*p* = 0.3077). A trend toward a higher incidence of extra-pulmonary visceral metastases was noted in the “burned-out” population (three patients vs. one patient in the “primary testicular” group), although such difference failed to prove statistically significant (*p* = 0.0632).

Data are summarized in Table 1.

**Table 1:** Patients' characteristics.

		**Primary testicular**	**Burned-out**	***p*-value**
Age (mean)		17 years 7 months	15 years	<0.00001
Systemic symptoms		0/11	4/5	0.0027
Serum markers mean (range)	aFP (ng/ml)	979 (2.05–4,604.3)	2,215 (2–10,997)	<0.00001
	hCG (mIU/ml)	530 (2–1,863.5)	14,831 (2–73,564.5)	<0.00001
	LDH (IU/L)	424 (238–620)	1,558 (372–4,052)	<0.00001
Stage				
	III	6/11 (55%)	1/5 (20%)	0.3077
	IV	5/11 (45%)	4/5 (80%)	
	Extra-pulmonary metastases	1/11 (9%)	3/5 (60%)	0.0632

### Primary Histology

All the patients in both groups had non-seminomatous tumors.

In the “primary testicular” group, all the patients underwent primary orchiectomy: eight patients had mixed histology (73%), two patients had yolk sac tumor (18%), and one patient had immature teratoma (9%).

In the “burned-out” group, diagnoses were made on biopsies from metastatic sites. Two patients had choriocarcinoma (40%), two patients had embryonal carcinoma (40%), and one patient had mixed histology (20%). Four patients underwent orchiectomy after the diagnosis of metastatic germ cell tumor had been established: in all four cases, histology revealed *in situ* neoplasia associated with areas of fibrosis and interstitial lymphoplasmacytic infiltrates. One patient affected by choriocarcinoma with multiple pulmonary metastases progressively developed respiratory distress and was considered unfit to undergo surgery.

A trend toward a higher incidence of choriocarcinoma and embryonal carcinoma in the “burned-out” group was noted, although it was not statistically significant (*p* = 0.0833).

### Toxicity

In the “primary testicular” population, two patients (18%) experienced “relevant toxicity” during treatment; both had grade 3 anemia and thrombocytopenia (18%), one had infection (9%), and one had renal and hepatic grade 3 toxicity associated with grade 3 arterial hypertension (9%).

In the “burned-out” population, four patients (80%) had “relevant toxicity;” all of them had grade 3 anemia and thrombocytopenia (80%), three had infectious complications (60%), two had simultaneous renal and pancreatic grade 3 toxicity (40%), two had neurological complications (20%), one had a severe hypertensive crisis (20%), one had chronic pulmonary toxicity (20%), one patient with massive pulmonary metastases had acute respiratory distress and pleural hemorrhage secondary to “choriocarcinoma syndrome” (14, 15) that required oxygen supplementation and chest tube placement (20%), and one had an allergic reaction (20%).

There were no toxicity-related deaths in both groups.

The “burned-out” population had a statistically significant higher incidence of relevant toxicity than “primary testicular” population (*p* = 0.0357) (Table 2).

**Table 2:** Toxicity and outcome results.

		**Primary testicular**	**Burned-out**	***p-*value**
Toxicity grade 3+		2/11 (18%)	4/5 (80%)	0.0357
Outcome mean (range)	EFS	38 m (5–119 m)	12 m (2–28 m)	0.0164
	OS	43 m (13–119 m)	15 m (6–28 m)	0.0299

### Outcome

The 11 patients in the “primary testicular” group were followed up for a mean time of 43 months (range 13 to 119 months). Five patients underwent surgery on residual secondary lesions after chemotherapy. At the last follow-up visit, 10 patients (91%) were alive and in complete clinical, radiological, and serological remission while one patient (9%) had radiological evidence of progression of disease; this patient decided to refer to a different institution for further treatment and was subsequently lost at follow-up. A second patient experienced relapse 29 months after the initial diagnosis and was successfully treated by salvage therapy with surgical resection of residual masses followed by high-dose chemotherapy and autologous stem cell transplantation. No deaths were recorded. Mean OS time for the “primary testicular” group was 43 months (range 13–119 months) and mean EFS was 38 months (range 5 to 119 months).

The five patients in the “burned-out” group were followed up for a mean time of 15 months (range 6 to 28 months). At the last follow-up, two patients (40%) affected by pure embryonal carcinoma were alive and in complete remission at 28 and 27 months, respectively; one of them underwent surgery on residual metastases after chemotherapy. One patient (20%) initially diagnosed with mixed tumor experienced clinical and radiological signs of progression of disease with negative serum markers at 3 months with histological evidence of mature teratoma, a condition described as “growing teratoma syndrome” (16); this patient underwent multiple debulking procedures of the residual lesions for symptomatic relief and was alive 7 months after initial diagnosis with progressive growing teratoma syndrome. Two patients (40%) affected by pure choriocarcinoma experienced progression of disease during treatment 2 months after the initial diagnosis; they both underwent surgery on secondary lesions for symptomatic relief and died due to progressive disease at 6 months. Mean OS time for the “burned-out” group was 15 months (range 6 to 28 months) and mean EFS was 12 months (range 2–28 months).

**Figure 3:**
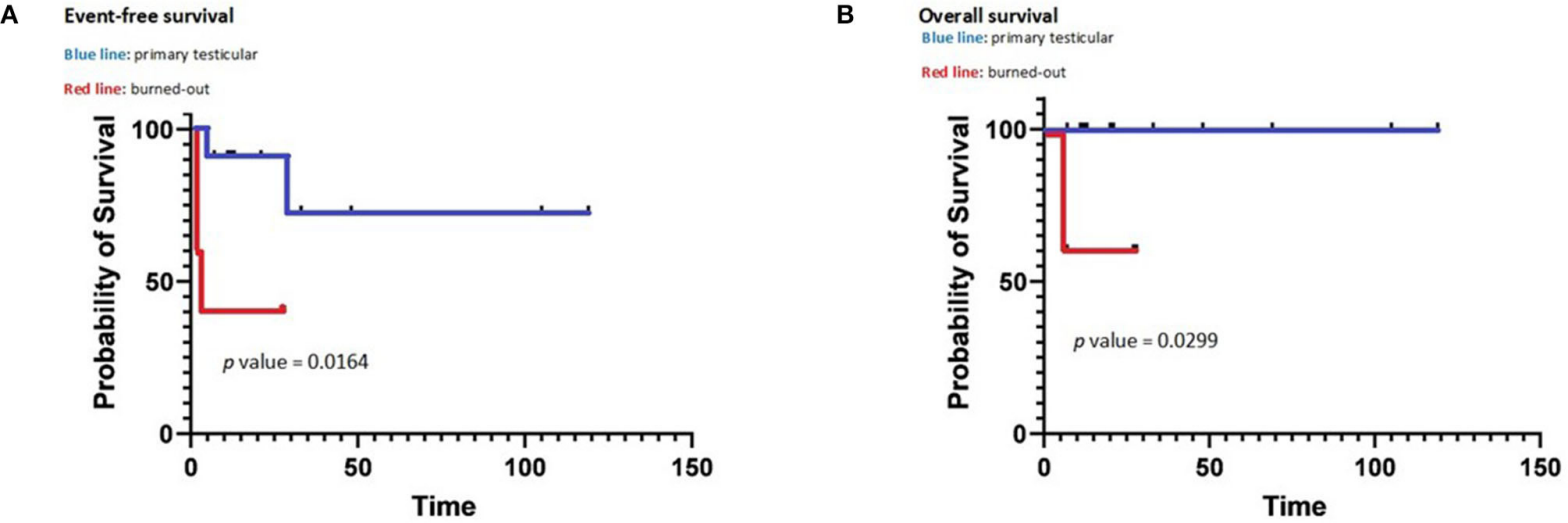
Event-free survival **(A)** and overall survival **(B)** of primary and burned out testicular tumors.

There was a statistically significant difference between “primary testicular” and “burned out” both in terms of OS (*p* = 0.0299) and EFS (*p* = 0.0164) as shown by the Kaplan–Meier plots (see Figure 3).

## Discussion

A burned-out testicular tumor is defined as spontaneous regression of a testicular germ cell tumor, which after metastatic spread manifests at its primary location as a scarring lesion with characteristic histological alterations (17). Such phenomenon was first described in 1927 by Prym in a patient with extragonadal choriocarcinoma (18) and has been reported both in adolescents and in adults (19). The histological features of burned-out testicular tumors were described by Azzopardi et al. in 1961; in this report, the authors described 17 adults who died due to metastatic choriocarcinoma and embryonal carcinoma and found a specific pattern of fibrous scarring associated with amorphous hematoxylin-staining deposits in dilated seminiferous tubules, mainly consisting in phospholipid, protein debris, and DNA, in some cases accompanied by small teratomatous structures and microscopic foci of seminoma (8). Subsequent studies have confirmed the characteristic microscopic appearance but have challenged the association of the “burn out” phenomenon with choriocarcinoma and embryonal carcinoma, describing “burned-out” tumors of all the histologic subtypes with a prevalence of seminoma, both pure and in association with other histotypes (10, 20).

The mechanism behind primary tumor regression has not been determined yet. One of the two main hypotheses postulate an immunological response mediated by cytotoxic T lymphocytes; the presence of a lymphoplasmacytic infiltrate and hemosiderin-containing macrophages in most histologic specimens from “burned-out” patients might support such hypothesis (10, 20). The second hypothesis postulates an ischemic response in the primary neoplasia, secondary to the blood supply deficit due to high metabolic rates and/or intermittent testicular torsion (21); such hypothesis is supported by the presence of testicular atrophy associated with scarring, reduced spermatogenesis, areas of necrosis, and coarse, large intratubular calcifications (11, 20).

Burned-out tumors clinically manifest with mass symptoms secondary to retroperitoneal, mediastinal, or supraclavicular lymph nodes or visceral metastases from germ cell tumors, in the absence of clinically apparent testicular masses (7, 22). To date, one case series has described the occurrence of weight loss and deep venous thrombosis associated with burned-out tumors (21), while two case reports have described the association with paraneoplastic neurological symptoms (i.e., ataxia and limbic encephalitis) (23, 24). Such clinical presentation and the presence of elevated LDH serum levels may contribute to initial misdiagnosis of lymphoproliferative disease in some patients (21) and provoke further delay in the correct diagnosis. Germ cell-specific serum markers, i.e., aFP and hCG, are often evaluated only after the correct diagnosis has been made on biopsy from metastatic sites and are elevated in case of non-seminomatous histology, especially when yolk sac or choriocarcinoma components are present, respectively (17).

Ultrasound scans in burned-out tumors typically show hypoechoic areas with irregular margins and heterogeneous adjacent parenchyma, diffuse microlithiasis and poor or absent vascular signals (25–27). Such findings, however, are non-specific, because also non-neoplastic lesions, such as hematomas or infarctions, may present with a similar sonographic pattern (28). One study by El Sanharawi et al. recently analyzed the vascularization of burned-out testicular tumor by dynamic contrast-enhanced magnetic resonance, demonstrating poor or absent enhancement in burned-out tumors (29), which is consistent with the typical histological appearance of fibrous scar and peripheral atrophy (8, 10).

To date, most papers about burned-out tumors consist in case reports or small case series; therefore, there are limited data about the outcome for burned-out patients. In the largest published series, tumor-related mortality ranges from 13% (22) to ~25% (17); however, these articles describe only adult patients and include both seminomatous and non-seminomatous tumors, which have a different prognosis (12). Moreover, there are no published data about treatment-related toxicity.

In the present work, all the patients in both groups are affected by stage III and stage IV non-seminomatous tumors. Patients in the “burned-out” group tended to be older than patients in the “testicular primary” group (mean age 17 years 10 months vs. 15 years 0 months); despite this difference, both populations are comprised in the range 13 to 19 years, which is reported to be the age range at highest risk of adverse events in testicular germ cell tumors (30). Histology and stage distribution did not differ significantly between the two groups and no patient had pre-treatment comorbidity. All the patients in both groups were treated according to the same protocol.

We may therefore assume that the two groups are comparable and that the differences in terms of clinical presentation, treatment-related toxicity, and outcome are attributable to the “burned-out” vs. “primary testicular” status.

All the patients in the “burned-out” population presented with a palpable metastatic mass, and four out of five patients also presented with systemic symptoms, i.e., weight loss, fever, and deep venous thrombosis, while none of the “primary testicular” patients in our series presented mass-related symptoms or systemic symptoms (*p* = 0.0027).

Serum markers at diagnosis were significantly higher in the “burned-out” population than in the “primary testicular” group: mean aFP 2,215 ng/ml (range 2–10,997 ng/ml) vs. 979 ng/ml (range 2.05–4,604.3 ng/ml), *p* < 0.00001; mean hCG 14,831 mIU/ml (range 2–73,564.5 mIU/ml) vs. 530 mIU/ml (range 2–1,863.5 mIU/ml), *p* < 0.00001; mean LDH 1,558 IU/L (range 372–4,052 IU/L) vs. 424 IU/L (range 238–620 IU/L), *p* < 0.00001.

The differences in clinical presentation and serum markers at diagnosis may be interpreted as a sign of higher tumor burden in the “burned-out” group; a different explanation could be a substantially different biological behavior of “burned-out” tumors compared to “primary testicular” tumors even in the face of similar disease stage. At the state of the art, there is poor evidence about the biological mechanism of the “burned-out” phenomenon and its clinical implications; further studies are needed to clarify this issue.

In the present case series, “burned-out” patients had a significantly higher incidence of relevant toxicity than “primary testicular” patients (*p* = 0.0357); such event is unexpected since both groups received the same therapeutic regimen and no patient had pre-existing comorbidities. One patient in the “burned-out” group experienced “choriocarcinoma syndrome,” a treatment-related complication that occurs in patients affected by choriocarcinoma with multiple pulmonary metastases at the beginning of chemotherapy, characterized by acute respiratory distress and pulmonary hemorrhage (14, 15). Apart from “choriocarcinoma syndrome,” the higher incidence of relevant toxicity observed in the “burned-out” population is unanticipated; it might be simply related to the small sample size, or it could be the result of a higher tumor burden in “burned-out” patients. Metastatic germ cell tumors and tumors with high LDH levels are at risk for tumor lysis syndrome during induction chemotherapy (31–34); “burned-out” patients present with both features and therefore might be at higher risk of renal toxicity. However, such mechanism needs further supporting evidence and would only explain renal toxicity. The treatment-related toxicity experienced by “burned-out” patients is currently an unexplained phenomenon; since the present paper is the first study that systematically addresses this issue, further studies are necessary in this field.

The present data show that “burned-out” patients have a worse outcome compared to “primary testicular” patients in terms of both OS (*p* = 0.0299) and EFS (*p* = 0.0164). To our knowledge, this is the first study to compare outcome between “burned-out” and “primary testicular” patients. Several factors may contribute to such difference. In the present series, “burned-out” patients had significantly higher levels of serum markers (i.e., aFP, hCG, and LDH) than “primary testicular” patients: elevated aFP, hCG, and LDH are known adverse prognostic factors in adults (12), although in pediatric patients, such association has been demonstrated for aFP only (4, 35). “Burned-out” patients also showed a trend toward a higher incidence of extra-pulmonary visceral metastases, although not statistically significant (*p* = 0.0632); the presence of such secondary lesions is a documented poor prognostic factor in adults (12) but not in the pediatric population at the state of the art.

The different clinical behavior could be explained by a biological difference between “burned-out” and “primary testicular” tumors. Further studies are needed to clarify this issue.

The present study has an obvious limitation in its retrospective nature. A second limitation is the small number of patients included in the study.

On the other hand, the two groups compared in this study showed a similar distribution in terms of age, stage, and histology; moreover, all the patients were treated according to the same protocol (5). It is reasonable to conclude that all the differences observed between the two groups may be secondary to the “burned-out” vs. “primary testicular” status.

In conclusion, “burned-out” testicular tumors seem to be a well-distinct clinical entity with a higher treatment-related toxicity and poorer prognosis compared to metastatic “primary testicular” tumors. Further studies are needed to clarify the “burned-out phenomenon” from a biological point of view and to identify more effective therapeutic strategies for these patients.

## Data Availability Statement

The raw data supporting the conclusions of this article will be made available by the authors, without undue reservation.

## Author Contributions

GP, AC, MD, RC, and AI contributed to conception and design of the study. FB and CM organized the database and revised the data. RA and FD revised the pathology specimens. PD revised radiology images. All authors contributed to manuscript revision and read and approved the submitted version.

## Conflict of Interest

The authors declare that the research was conducted in the absence of any commercial or financial relationships that could be construed as a potential conflict of interest.

## Publisher's Note

All claims expressed in this article are solely those of the authors and do not necessarily represent those of their affiliated organizations, or those of the publisher, the editors and the reviewers. Any product that may be evaluated in this article, or claim that may be made by its manufacturer, is not guaranteed or endorsed by the publisher.
